# Development of a quantitative Correlative Light Electron Microscopy technique to study GLUT4 trafficking

**DOI:** 10.1007/s00709-013-0597-5

**Published:** 2014-01-04

**Authors:** Lorna Hodgson, Jeremy Tavaré, Paul Verkade

**Affiliations:** 1 School of Biochemistry, Medical Sciences Building, University of Bristol, University Walk, Bristol, BS8 1TD UK; 2 School of Physiology and Pharmacology, Medical Sciences Building, University Walk, Bristol, BS8 1TD UK; 3 Wolfson Bioimaging Facility, School of Medical Sciences, University Walk, Bristol, BS8 1TD UK

**Keywords:** CLEM, Correlative microscopy, Intracellular transport, Insulin, Light microscopy, Electron microscopy, Immunolabelling

## Abstract

Correlative Light Electron Microscopy (CLEM) combines advantages of light microscopy and electron microscopy in one experiment to deliver information above and beyond the capability of either modality alone. There are many different CLEM techniques, each having its own special advantages but also its technical challenges. It is however the biological question that (should) drive(s) the development and application of a specific CLEM technique in order to provide the answer. Here we describe the development of a CLEM technique that is based on the Tokuyasu cryo immuno-gold labelling technique that has allowed us to quantitatively study GLUT4 trafficking.

## Introduction

### GLUT4

Our understanding of glucose homeostasis, insulin signalling and glucose transporter trafficking has increased significantly over the past few decades. Beginning with the initial observation that insulin treatment increases the number of glucose transporter sites within the plasma membrane (Cushman and Wardzala 1980) and leading on to the identification of a specific insulin-regulatable glucose transporter in muscle and adipose cells, now known as glucose transporter type 4 (GLUT4; James et al. 1988).

Research has focused on the localisation of GLUT4 in tissue and cells, the signalling events leading to the translocation of the transporter to the plasma membrane and the precise mechanisms and key proteins involved in these processes. However, despite significant advances in our knowledge of GLUT4 biology, much remains uncertain.

Immuno-electron microscopy studies have been an invaluable tool in intracellular trafficking studies (van Weering et al. 2010) as instanced by studying GLUT4 localisation and enabling resolution of different GLUT4 containing compartments within adipocytes and skeletal muscle. It has been demonstrated that in adipose tissue, the majority of GLUT4 is found in small tubular vesicular structures (50–80 nm in diameter), often occurring in clusters throughout the cytoplasm, with almost no transporter at the plasma membrane in the absence of insulin. Upon stimulation with insulin an increased labelling at the plasma membrane and in coated pits in the vicinity of the plasma membrane is observed, resulting in approximately 40 % of the total GLUT4 transporters relocating to the cell surface together with a concomitant loss of small GLUT4-containing vesicles from the interior of the cell (Malide et al. 2000; Slot et al. 1991). These small vesicles, termed GLUT4 storage vesicles (GSVs) are believed to be the principle donors responsible for insulin-stimulated GLUT4 delivery to the plasma membrane (Martin et al. 2000). Furthermore, whole mount quantitative immuno-electron microscopy of intracellular vesicles from adipocytes and whole cells has revealed that these GLUT4 positive vesicles are enriched in the SNARE (SNAP receptor) protein, VAMP2 (vesicle associated membrane protein 2) and IRAP (interleukin-1 receptor antagonist protein) and typically contain between 1 and 20 gold particles/vesicle (Martin et al. 2000; Ramm et al. 2000).

GLUT4 localisation has been studied in a variety of different insulin responsive tissues, such as white and brown adipose tissue (Slot et al. 1991), skeletal muscle (Ploug et al. 1998), and cardiac muscle (Slot et al. 1997). These tissues express high levels of GLUT4, with the majority of the transporter residing intracellularly in the absence of insulin. Most studies concerning GLUT4 trafficking however have utilised the 3T3-L1 adipocyte model system. 3T3-L1 fibroblasts can be differentiated into an adipocyte phenotype by treatment with a hormone cocktail. This established cell line shows similar insulin responsive characteristics, GLUT4 expression levels, morphology and protein markers to primary adipocytes, whilst being more suitable for manipulation and development of trafficking assays (Green and Kehinde 1975).

Much work on 3T3-L1 adipocytes has involved quantitative techniques using cells transiently expressing the reporter construct HA.GLUT4.GFP. This construct consists of a hemagglutinin (HA) epitope within the first extracellular loop of GLUT4 and a C-terminal GFP tag. The exofacial HA tag is only available for immunological detection upon GLUT4 translocation and insertion into the plasma membrane. In unstimulated cells, the construct concentrates in the juxtanuclear region with some staining in small punctuate structures in the cell periphery (Dawson et al. 2001). Stimulation with insulin causes a dramatic redistribution of the transporter to the plasma membrane resulting in a 7-fold increase in the surface to total ratio of HA.GLUT4.GFP (Zeigerer et al. 2002). Extensive characterisation of this construct reveals that it displays similar localisation, translocation and recycling kinetics to endogenous GLUT4, thus making it a valuable tool for studying GLUT4 trafficking (Dawson et al. 2001).

The HA.GLUT4.GFP chimera has been used in various quantitative techniques, including; endosomal ablation studies (Karylowski et al. 2004); calculation of exocytic and endocytic rate constants (Martin et al. 2006); confocal microscopy and live cell imaging (Eyster et al. 2006). Many of these methods involve using conventional fluorescence microscopes, however, it is impossible to distinguish between all GLUT4-containing compartments, in particular defining the GSVs, using this technique. This is in part due to the small size of GSVs and the resolution limitations of light microscopes and also due to the compact nature of the juxtanuclear region of adipocytes and muscle cells, resulting in high background fluorescence from neighbouring compartments, which interferes with the identification of single structures. Moreover, many of the fixation and extraction processes involved in fluorescence microscopy, required to allow antibody accessibility inevitably result in the loss of small vesicles, such as GSVs. Therefore an alternative high-resolution technique is needed in order to reliably study GSV localisation and trafficking of fluorescently tagged GLUT4.

### Correlative Light Electron Microscopy

Electron microscopy overcomes many of the limitations faced by conventional light microscopes. It achieves the highest degree of resolution and in addition, provides not only a more precise location of the protein of interest, but gives information on the ultrastructural environment and morphology of the compartment in which the protein is located (Slot and Geuze 2007; Brown et al. 2012). However, electron microscopy is not without fault, as it requires fixation and therefore does not allow the study of live events. In fact, no single microscopy technique is currently able to provide all the necessary information required to study trafficking of GLUT4 at the ultrastructural level.

Correlative Light Electron Microscopy (CLEM) exists in order to bridge the gap between light microscopy and electron microscopy. It combines the rapid sample screening and live cell imaging of light microscopy with detailed information on protein localisation and the 'reference space' offered by electron microscopes. CLEM typically involves imaging of fluorescently tagged proteins in either live or fixed cells at the light microscope level, followed by fixation, before retracing the same cell back in the electron microscope. However, this is somewhat challenging when you consider that neither fluorescent proteins nor gold particles are directly detectable in both light and electron microscopes, hence CLEM.

This problem can be circumvented by two methods, either by using two secondary probes — one that has a fluorochrome and the other a gold particle (Van Dam et al. 1991) (Sun et al. 1995), such as an Alexa Fluor secondary antibody and a colloidal gold marker, or by using a probe that contains both tags. A probe containing both tags is much more desirable, as it will produce lower background labelling and allow for true correlation of light and electron microscopy stages. Consequently, much research has focused on this issue, leading to the development of CLEM probes such as quantum dots (Giepmans et al. 2005) and the FlAsh/ReAsh system (Gaietta et al. 2002).

A major challenge is confronted when creating probes that are both electron dense and fluorescent is that the fluorescence signal can be quenched by fluorescence resonance energy transfer (FRET) onto the electron dense marker (Kandela and Albrecht 2007). In addition to this, artefacts can be created by aggregation and mis-targeting of the probes within the cell (Brown and Verkade 2010).

Consequently, one of the more widely used CLEM techniques does not require electron dense-fluorescent probes and instead relies upon fluorescent and/or endogenous proteins and immunolabelling after fixation. This is an adaptation of the Tokuyasu method of immunolabelling cryosections, originally developed in 1973 (Tokuyasu 1973; see also Slot and Geuze 2007). Briefly, cells are imaged either live using fluorescently tagged proteins before processing for electron microscopy and immunolabelling with antibodies against the fluorescent tag. Or alternatively, cells are fixed, embedded and sectioned as in conventional electron microscopy sample preparation, prior to labelling with antibodies against endogenous proteins, followed by labelling with a fluorescent secondary antibody coupled to a gold particle.

The correlation of immunofluorescence and electron microscopy of ultrathin Tokuyasu cryosections is a relatively simple process that has many advantages over other techniques. Firstly, it allows for the examination of multiple molecules, either endogenous or over expressed. Secondly, the fixation process retains good antigenicity whilst preserving cellular ultrastructure and finally, the signal/noise ratio of the fluorescence images is greatly improved as the imaged sections are no more than 150 nm in thickness (Takazawi and Robinson 2012).

The use of live GFP imaging followed by EM on the same sample was first applied in the study of the trafficking of vesicular stomatitis virus G protein (VSV-G) carriers through the Golgi towards the plasma membrane. It provided new insights into the precise details of Golgi transport intermediate formation and trafficking, revealing that the structures seen in the light microscope represented an almost entirely tubular–saccular structure, thus providing the first opportunity to combine information on cell structure with function (Polishchuk et al. 2000). Furthermore, Koster and Klumperman and our own group demonstrated that although fluorescent spots appear similar at the light microscopy level, they may represent entirely different compartments within the cell, highlighting once again the importance of correlative studies (Koster and Klumperman 2003; Hughes et al. 2009).

With the recent emergence of 3D high-data-output (Vicidomini et al. 2008), live-cell (Verkade 2008; Van Rijnsoever et al. 2008) and 4Pi CLEM (Perinetti et al. 2009), the future of CLEM, in particular Tokuyasu-based techniques looks set to remain a key player in the development of super-resolution imaging and in the study and understanding of cell structure, function and protein organisation.

### GLUT4 trafficking and CLEM

Many key questions still remain regarding GLUT4 trafficking, the nature of the GSVs and other GLUT4-containing compartments and the roles that insulin and various effector proteins play in GLUT4 translocation and recycling. It is therefore imperative that quantitative high-resolution techniques and assays, such as CLEM, are developed and applied to GLUT4 biology to allow further developments to be made. The HA.GLUT4.GFP construct has been extensively characterised and used in various assays and is thus a perfect candidate for use in CLEM studies.

This paper will therefore focus on the development of a suitable CLEM technique using the HA.GLUT4.GFP reporter construct, with the ultimate goal being to use this to study the effects that insulin and various other proteins may have on GLUT4 localisation and trafficking in adipocytes.

The aims of this paper are twofold:

1. Establish a CLEM assay to identify GLUT4 positive compartments in both the light and electron microscope and gain further insight into the ultrastructural nature of these compartments.
2. Quantify the effects of insulin on GLUT4 localisation and identify the compartments from which GLUT4 translocation occurs.

## Materials and methods

### Plasmids

#### pXLG_3_.HA.GLUT4.GFP

The plasmid, pQB125.HA.GLUT4.GFP was a gift from T. McGraw (Weill Cornell Medical College, New York, USA). It encodes full-length GLUT4 with a C-terminal GFP tag and a HA epitope tag in the first exofacial loop of GLUT4. In order to generate the pXLG_3_.HA.GLUT4.GFP plasmid, the nucleotide sequence encoding HA.GLUT4.GFP was amplified using the following primers; forward primer 5′-CGCTGGGATCCAAGAAGCTTATGCCGTCGG-3′ and reverse primer 5′-CGCTGGGATCCAAGAAGCTTATGCCGTCGG-3′. Primers with a 5′-*Nhe*I extension were designed to amplify HA.GLUT4.GFP. The PCR product and pXLG_3_ plasmid were digested with *Nhe*I, *Bam*HI and *Spe*I, *Bam*HI, respectively, and ligated together to create pXLG_3_.HA.GLUT4.GFP. Digestion resulted in excision of GFP from pXLG_3_ plasmid.

### Electron microscopy

#### Sample processing for electron microscopy

Cells were processed for electron microscopy largely as described by Tokuyasu (1973) and Slot and Geuze (2007). Briefly, 3T3-L1 adipocytes in 10-cm dishes were serum starved for 3 h and stimulated with or without insulin as required. Cells were fixed with 2 % (w/v) formaldehyde, 0.2 % (v/v) glutaraldehyde (Agar Scientific, UK) in 0.1 M phosphate buffer (81 mM Na_2_HPO_4_, 22 mM NaH_2_PO_4_, pH 7.4) at room temperature for 2 h. Cells were washed in PBS, quenched in 20 mM glycine for 5 min and detached by scraping in 1 % (w/v) gelatin in phosphate buffer, before centrifugation at 16,000×*g* for 5 s. Supernatants were removed and cell pellets embedded in pre-warmed 12 % (w/v) gelatin in phosphate buffer. After 10 min at 37°C, cells were centrifuged for 30 s, the supernatant removed and pellets placed on ice for 30 min to allow the gelatin to solidify. The solidified gelatin pellets were cut into 1-mm^3^ blocks and infiltrated overnight at 4°C with 2.3 M sucrose in phosphate buffer. Cell blocks were then mounted onto aluminium pins and transferred to liquid nitrogen until sectioning.

#### Cryosectioning

Ultrathin cryosections were cut (60–100 nm) using an EM UC6 microtome (Leica Microsystems) equipped with a FC6 Cryo unit (Leica Microsystems). Ribbons of sections were cut using a cryo-immuno 35° diamond knife (Diatome, Switzerland) and picked up in a 1 % methylcellulose/1.75 M sucrose solution before mounting onto carbon-coated formvar films on copper grids (Agar Scientific, UK). Sections were air-dried and stored at 4°C.

#### Immunolabelling

Grid-mounted sections were incubated on a drop of PBS for 30 min at 37°C to remove embedded gelatin and methylcellulose, before transferring to drops of 100 μM glycine in PBS and 0.1 % acetylated BSA (Aurion, The Netherlands) in PBS for 10 min. Grids were incubated in required primary antibody diluted in 0.1 % BSA/PBS for 1 h at room temperature before washing in 0.1 % BSA/PBS. After washing, grids were incubated in 5 or 10 nm Protein A Gold (Cell Microscopy Centre, Utrecht University Medical Centre, The Netherlands) diluted in 0.1 % BSA/PBS. Unbound gold was removed by washing grids in 0.1 % BSA/PBS and distilled water. Finally grids were counterstained with a solution of 0.3 % (w/v) uranyl acetate in 1.8 % methylcellulose for 5 min on ice. Grids were air-dried using the wire loop method.

#### Transmission electron microscopy (TEM)

Grids were examined on a Tecnai12 120 kV BioTwin Spirit transmission electron microscope (FEI Company, Eindhoven, Netherlands) equipped with a bottom-mount Eagle CCD camera (FEI company). Images were taken at 30,000× magnification.

#### Quantification and compartment identification

Whole cells were imaged and the gold labelling quantified by counting all gold particles located with 30 nm (Klumperman et al. 1998) of the compartments listed in Table 1.

**Table 1:** Electron microscopy compartment morphology

Compartment	Morphology
Plasma membrane	Limiting membrane of the cell
Clathrin-coated pits/vesicles at the plasma membrane	Clathrin-positive vesicles budding from/attached to the plasma membrane (clathrin labelled membranes were identified by an electron dense coat)
Vesicle/tubules near the plasma membrane	Vesicular–tubular structures within 200 nm of the plasma membrane and less than 200 nm in diameter
Early endosomes	A clear membranous vacuole greater than 200 nm in diameter containing fewer than eight intra-luminal vesicles
Late endosomes	Vacuoles greater than 200 nm in diameter with more than eight intraluminal vesicles
Lysosomes	Electron dense structures with or without internal vesicles or lamellar membranes
Golgi apparatus	A stack of three or more cisternae
TGN	Vesicular and tubular structures less than 200 nm in size, within 400 nm of the Golgi stack
Clusters of vesicles 50–100 nm in diameter	Greater than eight vesicles clustered together, within 500 nm of the nucleus
Vesicular/tubular elements in the juxtanuclear region of the cell	Structures smaller than 200 nm in diameter, located within 500 nm of the nucleus

Gold particles not associated with any of the above compartments were not included in quantitative analysis. Gold counts were expressed as a percentage of the total gold within each cell and normalised to the total membrane length of each organelle (μm).

### Correlative Light Electron Microscopy

3T3-L1 adipocytes stably expressing pXLG_3._HA.GLUT4.GFP were serum starved and stimulated with insulin as required before processing for electron microscopy as described in section 0. Then, 100-nm-thick cryosections were cut, picked up in a drop of 1 % methylcellulose/1.75 M sucrose and mounted onto formvar film and carbon coated copper finder grids (Agar, Scientific, UK). Grids were then incubated on a drop of PBS at 37 ºC for 30 min and counterstained with DAPI for 5 min. Grids were mounted between glass slides and coverslips on a drop of 50 % glycerol in PBS. Cells expressing the pXLG_3_.HA.GLUT4.GFP construct were identified using an AM total internal reflection fluorescence multi-colour system (Leica Microsystems) in widefield mode attached to a DMI 6000 inverted epifluoresence microscope (Leica Microsystems). Finder grids were used to provide a reference point for expressing cells. Grids were removed from glass slides and washed with water before immunolabelling as described. Cells expressing pXLG_3_.HA.GLUT4.GFP were identified using an anti-GFP antibody and Protein A gold. Cells identified by fluorescence microscopy were imaged in TEM and the locations of gold particles within each cell were counted.

## Results

### Development and characterisation of a pXLG_3_.HA.GLUT4.GFP cell line

The HA.GLUT4.GFP chimera has been extensively characterised and used in various quantitative assays in the study of GLUT4 trafficking in 3T3-L1 adipocytes (Karylowski et al. 2004; Zeigerer et al. 2002). In these studies, a variety of methods were employed in order to introduce the HA.GLUT4.GFP DNA into adipocytes; including electroporation (Quon et al. 1993) and transduction using adenovirus (Orlicky and Schaack 2001). However, these methods have limitations and neither result in especially high transfection rates, as adipocytes are not particularly susceptible to most gene transfer methods. And although CLEM is able to deal with very low transfection efficiencies very well (one of the highlights of CLEM is actually the ability to find/retrace the one transfected cell amongst hundreds of untransfected cells) in order to obtain quantitative data higher transfection levels were for this particular application desirable.

Carlotti and colleagues described a new method of genetic modulation of adipocytes using HIV-1 derived lentiviral vectors. They showed that both differentiated adipocytes and undifferentiated preadipocytes can be successfully transduced with lentiviral vectors, resulting in a roughly 95 % transfection efficiency and no significant toxicity effects, thus demonstrating a much more suitable method of gene transfer in the 3T3-L1 model system (Carlotti et al. 2004).

3T3-L1 adipocytes transduced with pXLG_3_.HA.GLUT4.GFP were examined in order to determine firstly, whether infection with lentivirus and subsequent generation of a pseudo stable cell line, altered the localisation of GLUT4 and the expression of key proteins involved in GLUT4 trafficking and secondly, whether these cells showed similar trafficking properties to electroporated cells. 3T3-L1 fibroblasts were transduced with a multi-attenuated HIV-1 derived lentivirus, encoding the pXLG_3_.HA.GLUT4.GFP reporter construct, before subculturing and finally differentiation into quiescent mature adipocytes.

**Fig. 1:**
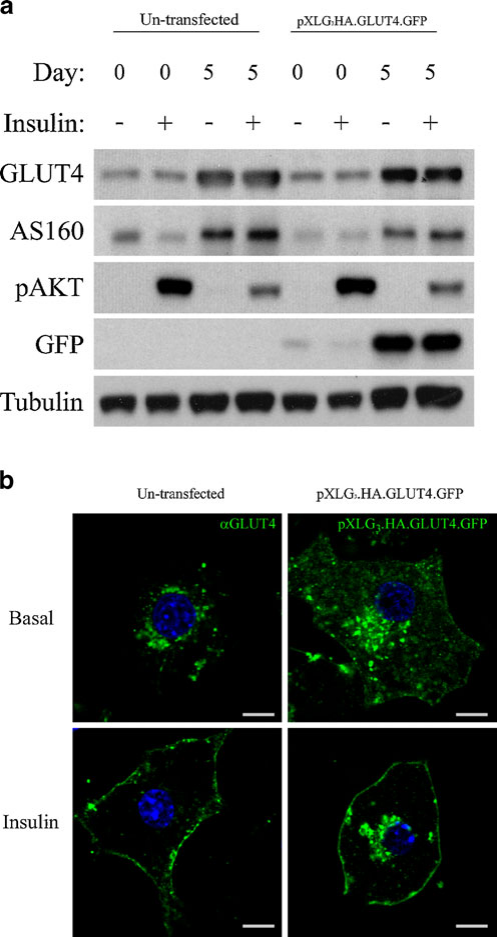
Comparison of protein expression, differentiation and GLUT4 localisation in un-transfected and lentivirally transduced 3T3-L1 adipocytes. **a** 3T3-L1 fibroblasts and adipocytes un-transfected or transduced with pXLG3.HA.GLUT4.GFP were serum starved for 3 h and treated with or without insulin (87 nM) for 20 min prior to lysis in 0.1 % Triton buffer. Samples were separated by SDS-PAGE and analysed by Western blotting using GLUT4, AS160, p-AKT(S473), GFP and Tubulin (loading control) antibodies. **b** 3T3-L1 adipocytes were serum starved and stimulated in the presence and absence of insulin and fixed in 4 % PFA for 20 min. Un-transfected cells were immunostained with antibodies against endogenous GLUT4 followed by Alexa Fluor 488 secondary antibodies, whilst adipocytes stably expressing pXLG3.HA.GLUT4.GFP were analysed in fixed cells based on their GFP fluorescence. Scale bar = 8 mm

The expression levels of GLUT4, AS160, pAkt and GFP were examined by Western blotting (Fig. 1a). The data reveals that the levels of endogenous GLUT4 and AS160 increased dramatically upon differentiation into mature adipocytes (day 5) and importantly, transduction of cells with lentivirus did not significantly alter this up-regulation. In addition, the levels of pXLG_3_.HA.GLUT4.GFP, determined by blotting with an anti-GFP antibody, showed a similar increase in expression. The levels of these endogenous and over expressed proteins remained unchanged upon stimulation with insulin. However, stimulation with insulin did increase the amount of phosphorylated Akt in both adipocytes and fibroblasts, with higher levels seen in the latter. No difference was observed between the stable cell line and un-transfected cells, demonstrating effective insulin signalling and activation of Akt within the lentivirally transduced cell line.

These findings were further supported by confocal microscopy analysis of the cells in basal and insulin-stimulated conditions (Fig. 1b). In the basal state, both endogenous and over expressed GLUT4 were concentrated in the juxtanuclear region with some small punctate staining throughout the cell periphery (Fig. 1b). Upon stimulation with insulin an increased amount of GLUT4 was observed at the plasma membrane. Furthermore, no differences in the localisation of endogenous GLUT4 and pXLG_3_.HA.GLUT4.GFP were found. This data is consistent with the previous findings of Carlotti and coworkers (2004). They demonstrated that lentiviral-mediated transfer of DNA into fibroblasts is stable and efficient and most importantly, has no affect on insulin-stimulated glucose uptake and adipocyte differentiation (Carlotti et al. 2004).

**Fig. 2:**
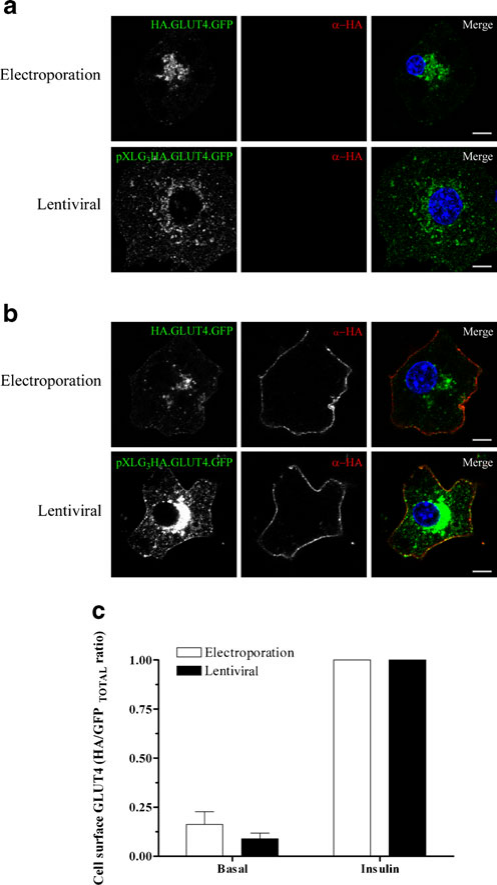
Comparison of GLUT4 localisation and translocation in 3T3-L1 adipocytes transiently transfected by electroporation or stably transduced by lentivirus. 3T3-L1 adipocytes stably expressing pXLG3.HA.GLUT4.GFP or transiently expressing HA.GLUT4.GFP after electroporation were serum starved for 3 h and stimulated in the absence (**a**) or presence (**b**) of insulin (87 nM) for 20 min. Fixed, non-permeabilised cells were stained using a HA antibody followed by labelling with a fluorescently tagged Alexa Fluor 633 secondary antibody. Images of expressing cells were acquired using a confocal microscope and quantified as described in Materials and methods. Scale bar = 10 mm. **c** Average surface-to-total (HA/GFP) ratio of GLUT4 in basal and insulin-stimulated conditions. The data is normalised to the insulin condition and represents three independent experiments. Error bars = SEM

A comparison of the cell surface levels of GLUT4 was made between electroporated and stably expressing adipocytes in order to determine whether transduction with lentivirus altered GLUT4 trafficking; in particular, the translocation of the transporter to the plasma membrane in response to insulin stimulation. 3T3-L1 adipocytes stably expressing pXLG_3_.HA.GLUT4.GFP, or cells transiently over expressing the HA.GLUT4.GFP reporter chimera after electroporation were treated with insulin and fixed. The amount of cell surface GLUT4 was detected using an HA antibody in un-permeabilised cells and the degree of GLUT4 translocation in response to insulin was then calculated from the surface-to-total ratio (HA/GFP). Under basal conditions, HA.GLUT4.GFP from electroporated and lentivirally transduced cells was almost entirely intracellular, concentrating around the nucleus and in diffuse punctae located throughout the cytoplasm (Fig. 2a). Although some vesicles appeared to be within close proximity of the plasma membrane in the GFP channel, little or no HA staining was observed at the cell surface. In contrast, when stimulated with insulin, a dramatic redistribution of the transporter to the plasma membrane was seen, represented by rings in the HA, and to a lesser extent, in the GFP channel (Fig. 2b). Quantification of the surface-to-total ratio reveals a 7- and 11-fold effect of insulin in electroporated cells and stable cell lines, respectively.

Transfection rates were also calculated to determine the efficiencies of both methods. Transduction with lentivirus resulted in roughly 80 % of cells expressing pXLG_3_.HA.GLUT4.GFP, whereas electroporation was significantly lower, with less than 20 % of cells expressing the construct.

In summary, lentiviral transduction of fibroblasts and the subsequent differentiation into stably expressing adipocytes is an effective method of gene transfer that does not alter differentiation, protein expression, localisation or translocation of GLUT4. Furthermore, the pXLG_3_.HA.GLUT4.GFP, 3T3-L1 adipocyte stable cell line is a suitable model system for studying GLUT4 trafficking.

### Adapting the Tokuyasu method for quantitative Correlative Light Electron Microscopy

Despite higher levels of transfection being achieved using lentiviral mediated transduction of cells, not all cells express pXLG_3_.HA.GLUT4.GFP, which is therefore problematic when trying to study GLUT4 localisation and trafficking at the electron microscopy level. It is essential to ascertain whether a cell is expressing the required protein before examining cells under the TEM.

CLEM is therefore an invaluable tool to allow the high-resolution study of the HA.GLUT4.GFP reporter chimera. The light microscopy stage of CLEM is required to find fluorescent cells in order to reduce the time involved in searching at the TEM level.

In addition, CLEM allows information about cell morphology and ultrastructure to be gathered, a key limitation of conventional fluorescence microscopy techniques. Henceforth, in order to study GLUT4 localisation and develop subsequent trafficking assays using the pXLG_3_.HA.GLUT4.GFP stable cell line, the Tokuyasu method of CLEM was established and used to validate previous observations using light microscopy and the HA.GLUT4.GFP reporter chimera.

**Fig. 3:**
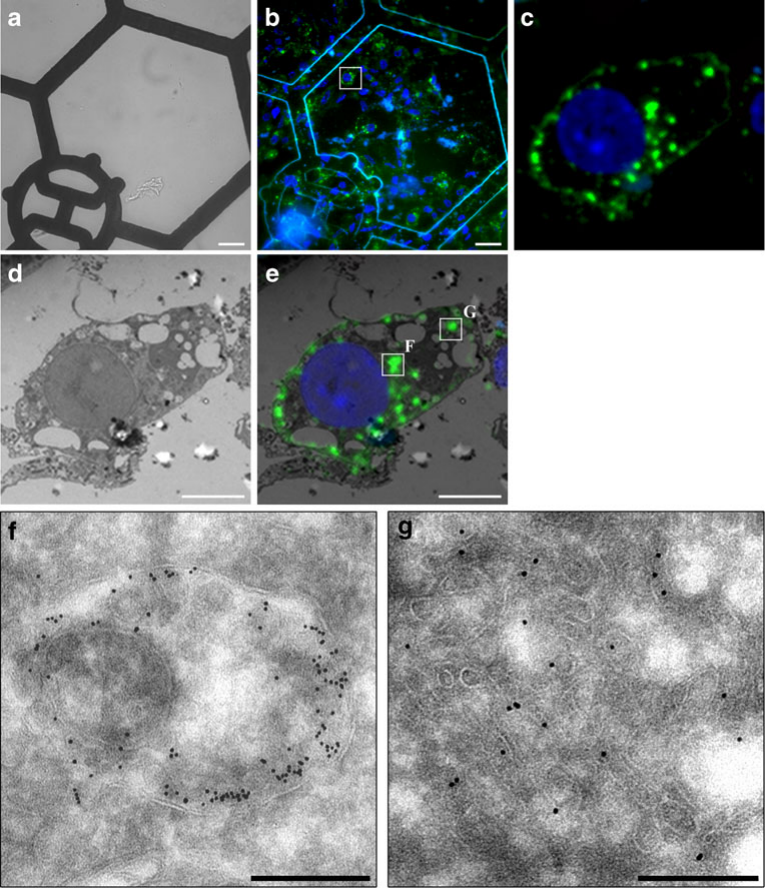
Correlative Light Electron Microscopy of 3T3-L1 adipocytes. 3T3-L1 adipocytes stably expressing pXLG3.HA.GLUT4.GFP were serum starved, stimulated with insulin and fixed before embedding in gelatin. Cryosections were cut (100 nm thick) and mounted onto coated finder grids. **a** LM image of finder grids at ×40 magnification. **b** LM of ribbon of sections with DAPI staining of nucleus. Scale bars = 38 mm. **c** Higher magnification of boxed area in **b. d** TEM low magnification image of same cell from LM. **e** Overlay of TEM and LM images. Scale bar = 5 mm. **f**, **g** Higher magnification TEM images of *boxed areas* from **e**. Immuno-gold labelling with an anti-GFP antibody and 10 nm Protein A gold. Scale bar = 200 nm

3T3-L1 adipocytes expressing pXLG3.HA.GLUT4.GFP were serum starved, stimulated with insulin and fixed before processing for CLEM. The procedure consists of the following stages: (1) processing of samples for standard electron microscopy; embedding in gelatine and cryosectioning; (2) imaging at the light microscopy level; (3) immunolabelling; (4) TEM imaging, (5) retracing and quantification. Figure 3 shows the final stages of the CLEM process, demonstrating the ability of the technique to image the same cell at the light microscope, based on its fluorescence (Fig. 3a,b,c) and in the electron microscope, based on gold labelling (Fig. 3d,f,g). The images taken at higher magnification in electron microscope, shown in Panel F and G highlight the inadequacies and resolution limitations of light microscopes. The areas appear as considerable bright fluorescent spots, suggesting that GLUT4 is localised to a single, large compartment; however, when examined at the electron microscopy level, it is revealed that these regions firstly may correspond to two completely different compartments and secondly, the compartments may represent a collection of vesicles and tubules, rather than a large single structure.

### Subcellular localisation of pXLG_3_.HA.GLUT4.GFP

Immuno-electron microscopy studies have proven to be imperative in identifying GLUT4 compartments within adipocytes. Endogenous GLUT4 has been shown to localise to a range of organelles, including the TGN, Golgi, endosomes, plasma membrane and lastly clusters of tubular vesicular structures within the cytoplasm of the cell, postulated to be the GSVs (Blok et al. 1988; Slot et al. 1991). In order to determine whether pXLG_3_.HA.GLUT4.GFP distributes to the same compartments as described previously, a detailed morphological analysis of GLUT4 positive organelles was performed.

**Fig. 4:**
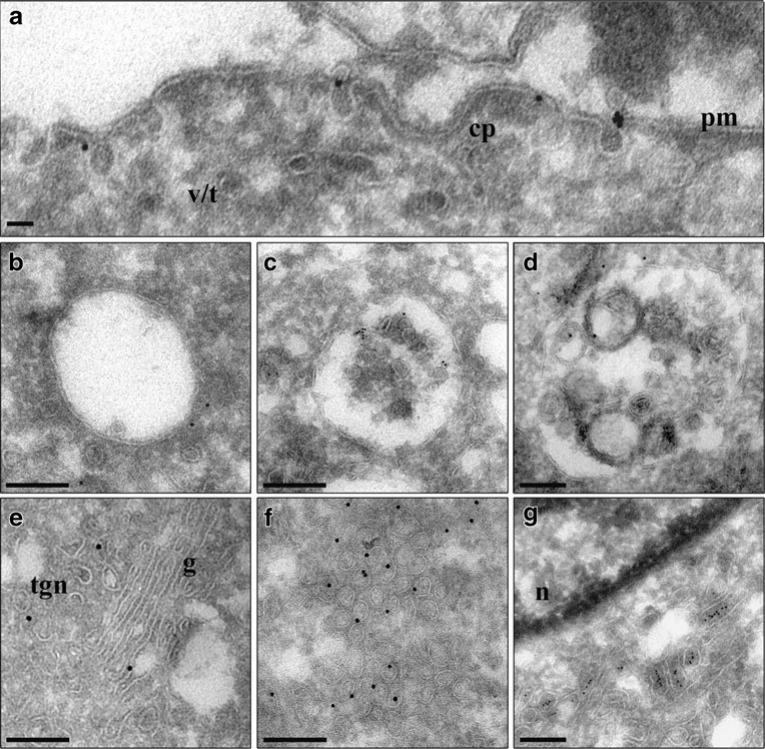
Transmission electron microscopy of intracellular compartments in 3T3-L1 adipocytes. 3T3-L1 adipocytes expressing pXLG3.HA.GLUT4.GFP were fixed and processed for CLEM. Sections were labelled with an anti-GFP antibody and 5 or 10 nm Protein A gold. Representative TEM images of intracellular compartments positive for pXLG3.HA.GLUT4.GFP and identified based on organelle morphology were taken. **a** Plasma membrane (*pm*), coated pits/vesicles at PM (*cp*) and vesicles/tubules near the PM (*v*/*t*). **b** Early endosomes. **c** Late endosomes. **d** Lysosomes. **e** Golgi (*g*) and TGN (*tgn*). **f** Clusters of vesicles. **g** Vesicles/tubules in juxtanuclear region. Scale bar = 200 nm

3T3-L1 adipocytes were prepared for CLEM and whole cells were examined under the TEM and the labelling pattern of GLUT4 was assessed. Consistent with earlier studies, GLUT4 was found to localise to the plasma membrane, endosomes (late and early), Golgi, TGN and clusters of small vesicles. The clusters consisted of a large group of homogeneous vesicles, typically less than 100 nm in diameter, located around the nucleus and stained with multiple gold particles, suggesting a large amount of GLUT4 labelling in these structures. Taken together this evidence supports previous findings that these structures most likely correspond to the GLUT4 storage pool and the GSVs. In addition to the compartments listed above, GLUT4 was also seen in lysosomes and a set of vesicles and tubules located in the juxtanuclear region of the cell unlike any other organelle or structure described (Fig. 4). The latter may represent an undefined GLUT4 compartment, a subset of vesicles or a subdomain located within the TGN (Shewan et al. 2003). Alternatively, it may correspond to a set of transport intermediates operating between the Golgi, TGN and GSVs.

Under basal conditions in 3T3-L1 adipocytes, the majority of GLUT4 labelling is intracellular, with roughly a quarter residing on small vesicles present in the cytoplasm. According to Blok and coworkers over half of the total endogenous GLUT4 labelling in the basal state is found on tubulovesicular structures, most likely representing the TGN and vesicles associated with the TGN, termed CURL (compartment of uncoupling receptors and ligands) (Blok et al. 1988). A quantitative analysis of the labelling densities of pXLG_3_.HA.GLUT4.GFP across each organelle was therefore performed and compared to that of endogenous GLUT4. This approach was taken in order to firstly determine the compartments in which pXLG_3_.HA.GLUT4.GFP was most abundant and whether this supports previous findings from tissues and cells and, secondly, whether the labelling distribution of over-expressed GLUT4 correlated with that of the endogenous protein.

**Fig. 5:**
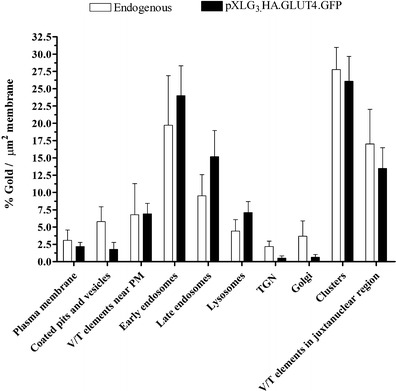
Quantification of pXLG3.HA.GLUT4.GFP localisation in 3T3-L1 adipocytes. Un-transfected 3T3-L1 adipocytes and cells stably expressing pXLG3.HA.GLUT4.GFP were serum starved for 3 h, fixed and embedded in gelatin. Cryosections were cut (100 nm) and un-transfected cells were stained with an anti-GLUT4 antibody followed by 10 nm Protein A gold and pXLG3.HA.GLUT4.GFP expressing cells were first imaged by LM, before staining with an anti-GFP antibody and 10 nm gold. Whole cells were imaged under TEM and gold labelling assigned to compartments as stated in Table 1. Gold counts were expressed as % of total gold per cell normalised to membrane length. (Endogenous: *N* = 15 cells, *n* = 450 gold particles; pXLG3.HA.GLUT4.GFP: *N* = 15, *n* = 532.) Error bars = SEM

3T3-L1 adipocytes un-transfected or expressing pXLG_3_.HA.GLUT4.GFP, were processed for standard Tokuyasu electron microscopy or CLEM, respectively. Sections were labelled with an anti GFP or GLUT4 antibody and protein A gold, prior to imaging under the TEM. Gold particles were assigned to compartments and the labelling densities were calculated as the percentage of immuno-gold label per membrane length. As shown in Fig. 5, the majority of the labelling for both conditions was found within vesicle clusters (~25 %), early endosomes (~20 %) and vesicular tubular elements in the juxtanuclear region (15 %). Less than 5 % of the total GLUT4 was found at the plasma membrane, thus supporting previous studies showing that almost all GLUT4 is retained intracellularly in unstimulated cells (Slot et al. 1991). Conversely, few gold particles for both endogenous and pXLG_3_.HA.GLUT4.GFP expressing cells were observed in the TGN and Golgi.

Taken together, these data demonstrate the usefulness of CLEM in distinguishing between different GLUT4 positive compartments based on their morphology and gold labelling. In addition it also reveals that cells stably expressing pXLG_3_.HA.GLUT4.GFP show similar localisation patterns to endogenous GLUT4, with most of the transporter residing intracellularly in a specialised storage pool.

### Insulin promotes the translocation of pXLG_3_.HA.GLUT4.GFP to the plasma membrane

**Fig. 6:**
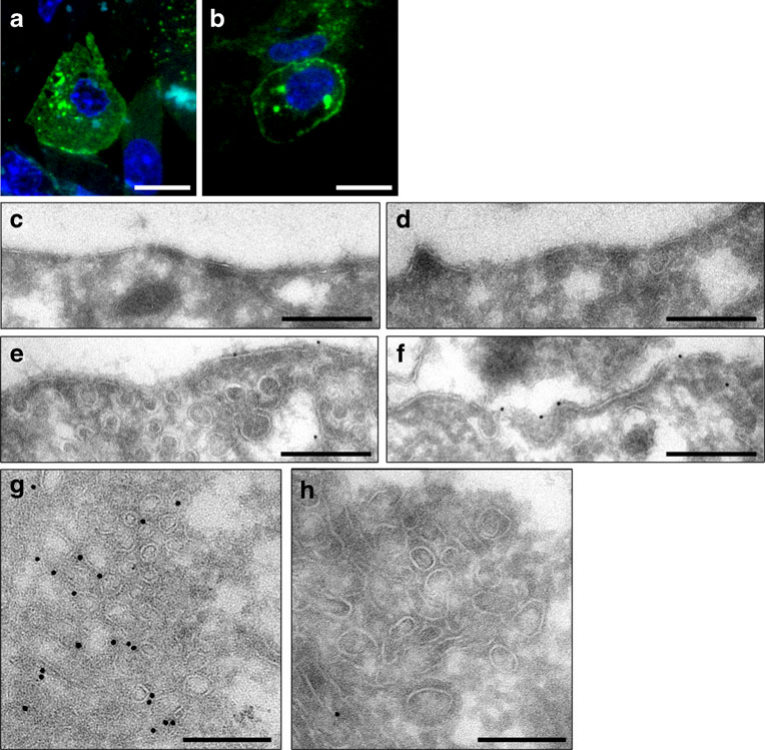
Insulin promotes the translocation of pXLG3.HA.GLUT4.GFP to the plasma membrane. 3T3-L1 adipocytes expressing pXLG3.HA.GLUT4.GFP were serum starved, stimulated without (**a**, **c**, **d**, **h**) or with (**b**, **e**, **f**, **g**) 87 nM insulin and fixed. Samples were prepared for CLEM, imaged in LM (**a**, **b**), scale bar = 15 mm, and sections were labelled with an anti-GFP antibody and 10 nm Protein A gold. Representative TEM images of plasma membrane in presence and absence of insulin (**c**, **d**, **e**, **f**). Scale bar = 500 nm. TEM image of vesicle clusters (**g**, **h**). Scale bar = 200 nm

The localisation of pXLG_3_.HA.GLUT4.GFP after stimulation with insulin was then assessed to determine the differences in GLUT4 labelling in basal and insulin stimulated conditions. 3T3-L1 adipocytes stably expressing pXLG_3_.HA.GLUT4.GFP, treated with or without insulin, were examined and the percentage gold labelling within each compartment, as described in Materials and methods, was calculated and normalised to total membrane length. Figure 6 reveals an absence of gold particles in the plasma membrane of unstimulated cells (Fig. 6c and d) and a large amount of labelling of clusters (Fig. 6h). Upon stimulation with insulin, fewer gold particles were found within the clusters of vesicles (Fig. 6g) and instead the majority of the protein was located at the cell surface (Fig. 6e and f). In addition, an increase in labelling of vesicles within the plasma membrane and in pits associated with the membrane is observed, and upon closer examination it appears that a greater number of vesicles, both positive and negative for GLUT4, are located within the vicinity of the plasma membrane (Fig. 6e). This therefore suggests an increase in exocytosis of GLUT4 as well as other endocytic cargo.

**Fig. 7:**
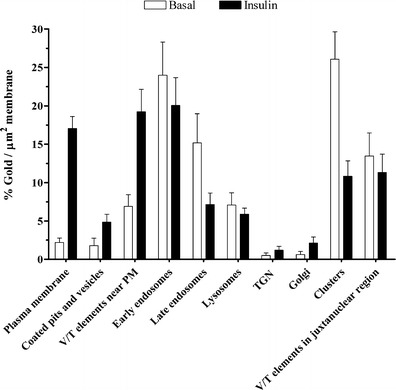
Insulin promotes the bulk recruitment of pXLG3.HA.GLUT4.GFP to the plasma membrane from vesicle clusters and tubular vesicular elements within the juxtanuclear region of the cell. Quantitative analysis of 3 T3-L1 adipocytes expressing pXLG3.HA.GLUT4.GFP in the presence and absence of insulin taken from two independent experiments. Gold labelling is expressed as % of total gold/membrane length. (Basal: *N* = 15 cells, *n* = 498 gold particles; insulin: *N* = 15, *n* = 592.) Graph represents mean ± SEM

Quantification of GLUT4 localisation in insulin and basal conditions supports these initial observations (Fig. 7). A 7-fold enrichment of cell surface GLUT4 and a 3-fold increase in GLUT4 in vesicles and tubules near the plasma membrane was observed in the insulin-stimulated state. This was accompanied by a 3-fold decrease in labelling of clusters and a 3-fold decrease in the amount of GLUT4 within the endosomes. Taken together, this data supports the evidence suggesting that GLUT4 translocates to the plasma membrane from a specialised storage pool consisting of a collection of small vesicles, roughly 70 nm in diameter.

## Discussion

This paper details the development and characterisation of a stable pXLG_3_.HA.GLUT4.GFP 3T3-L1 fibroblast cell line and its subsequent use in a quantitative CLEM assay to study GLUT4 localisation in adipocytes at the ultrastructural level.

### Generation and characterisation of pXLG_3_.HA.GLUT4.GFP stable cell line

The 3T3-L1 adipocyte model system combined with the HA.GLUT4.GFP reporter construct has proven an invaluable tool in the advancement of GLUT4 biology. Henceforth, a stable fibroblast cell line expressing a lentiviral version of this reporter protein, pXLG_3_.HA.GLUT4.GFP was generated. When transduced with lentiviral vectors encoding pXLG_3_.HA.GLUT4.GFP, fibroblasts showed stable incorporation and expression of the reporter construct. In addition, no effects on the ability of the fibroblasts to differentiate into adipocytes were observed, as determined by the presence of multiple fat droplets within the cell and by the clear up-regulation of classic adipocyte markers such as GLUT4 and AS160 (Fig. 3).

Surprisingly, the expression of the pXLG_3_.HA.GLUT4.GFP reporter construct was dramatically increased upon differentiation of cells into adipocytes when measured using a GFP antibody (Fig. 3). When quantified by densitometry, it was revealed that the expression levels increased approximately 15-fold, when normalised to the tubulin levels. This suggests that the lentiviral construct under the control of the CMV promoter is also up-regulated upon differentiation into adipocytes.

No differences in GLUT4 localisation at the light microscope level were apparent in pXLG_3_.HA.GLUT4.GFP expressing cells. In the basal state, pXLG_3_.HA.GLUT4.GFP displayed an intracellular localisation, concentrated in the juxtanuclear region. Upon stimulation with insulin, a large proportion of the transporter redistributed to the plasma membrane, with the remainder localising to the juxtanuclear region (Figs. 1b and 2a,b).

A comparison between electroporated and lentiviral transduced cells was made and when cell surface HA levels were examined, insulin was shown to promote the translocation of both pXLG_3_.HA.GLUT4.GFP and HA.GLUT4.GFP expressing cells by 11- and 7-fold, respectively (Fig. 2). The differences in the fold effect of insulin seen are a consequence of higher basal GLUT4 cell surface levels in electroporated cells and not due to reduced activation of pAkt in the stimulated state or conversely, increased pAkt activation in the basal state (Fig. 1). Overexpression of GLUT4 has previously been suggested to saturate the basal retention mechanisms and thus increase the amount of GLUT4 in the plasma membrane and increase the glucose uptake into unstimulated adipocytes (Carvalho et al. 2004).

Muretta and coworkers (2008) demonstrated that cells expressing HA.GLUT4.GFP after lentiviral infection do not display high enough expression levels to saturate the retention mechanism and as a consequence all adipocytes exhibited low surface-to-total ratios in the basal state. Therefore, the differences in the fold effect of insulin seen in this paper are most likely an artefact of the extreme stress placed upon the cell and/or the high protein expression levels, saturating the interactions with the basal retention machinery caused by electroporating the cells, rather than alterations made by lentiviral transduction. In addition, Carlotti et al. (2004) showed that modification of preadipocytes using lentivirus had no affect on basal or insulin stimulated glucose uptake. Taken together, this data therefore suggests that the generated stable pXLG_3_.HA.GLUT4.GFP cell line is a suitable model in which to study GLUT4 trafficking.

### Using quantitative correlative light electron microscopy to study pXLG_3_.HA.GLUT4.GFP trafficking in 3T3-L1 adipocytes

Examination of the subcellular distribution of HA.GLUT4.GFP reveals a largely juxtanuclear localisation with some staining of vesicular structures in the cell periphery (Dawson et al. 2001). A CLEM technique was developed and used to identify GLUT4 positive compartments within this juxtanuclear region and throughout the entire cell. The data revealed that the juxtanuclear staining of HA.GLUT4.GFP and endogenous GLUT4, seen in light microscopy imaging, corresponded to multiple compartments, including endosomes, clusters of small vesicles and collections of tubular vesicular elements, with the diffuse punctate staining distributed throughout the cytoplasm, most likely representing endosomal structures. When quantified, the majority of the immuno-gold staining in unstimulated cells corresponded to clusters of vesicles located within the interior of the cell. These structures represented a collection of multiple vesicles that were small, typically between 50 and 100 nm in diameter and were often situated within the juxtanuclear region of the cell. Stimulation with insulin resulted in a decrease in labelling of these vesicles, suggesting a translocation of GLUT4 from within this compartment to the plasma membrane.

Previous immuno-electron microscopy studies of adipose tissue and 3T3-L1 adipocytes have revealed a population of small tubular vesicular structures, 50–70 nm in diameter, enriched in GLUT4, that represent the insulin responsive compartment (Martin et al. 2000; Slot et al. 1991). Treatment with insulin results in a reduction of the amount of GLUT4 labelling these structures (Blok et al. 1988; Slot et al. 1991). Furthermore, biochemical fractionation data shows that a large proportion (~60–75 %) of cellular GLUT4 is present on small (80–100S) vesicles from low density microsomal membranes (Kandror et al. 1995). In addition, these membranes contain the insulin-regulated amino peptidase (IRAP) (Keller et al. 1995) and the v-SNARE, VAMP2 (Cain et al. 1992). The data, therefore, suggests that the clusters of vesicles seen in Figs. 4 and 5, most likely represent insulin-responsive GSVs.

Closer examination of fractionation data of the small vesicular structures, believed to be GSVs, revealed two populations of these vesicles, marked by the presence and absence of the synaptogyrin homologue, cellugyrin (Kupriyanova and Kandror 2000). Cellugyrin positive vesicles rapidly label with recycling markers such as transferrin and mannose-6-phosophate receptor, are negative for endosomal markers such as EEA1 and Rab11 and most interestingly, do not translocate to the plasma membrane in response to insulin (Kupriyanova et al. 2002). Evidence suggests that this subpopulation of vesicles may represent a set of ubiquitous vesicles derived from the TGN and endosomes, potentially involved in transport of cargo between early and recycling and endosomes and the TGN (Belfort and Kandror 2003). Interestingly, the subset of vesicles identified in this paper, characterised as, vesicular/tubular elements in the juxtanuclear region of the cell, are typically less than 150 nm in diameter and contain a large portion of GLUT4 (~15 %). Furthermore, the amount of GLUT4 within this compartment remains unchanged after stimulation with insulin, suggesting that these vesicles may represent the cellugyrin-positive subset of vesicles described previously.

Whether these vesicular tubular elements within the juxtanuclear region of the cell represent a specialised compartment and what role they may play in GLUT4 sorting and recycling remains to be determined. In addition, ascertaining whether the clusters of vesicles identified are GSVs and whether they represent the insulin responsive compartment is another important unanswered question. Defining these compartments further has been hampered by the availability of antibodies against endogenous GLUT4 vesicle markers, such as IRAP, VAMP-2 and cellugyrin.

Both the GSVs and vesicular/tubular elements in the juxtanuclear region are less than 200 nm in size and therefore are diffraction limited and not readily resolved using fluorescence microscopes, hence it is imperative that super resolution imaging techniques combined with electron microscopy, such as 4Pi CLEM and live-cell CLEM, be developed in order to examine these structures further. Developments in these technologies may provide insights into determining the precise nature and role of these small GLUT4-containing vesicles; in particular, whether GSVs translocate to the plasma membrane directly from the storage pool, or if they traffic indirectly via the endocytic network.

Roughly, 35 % of the total GLUT4 labelling is found within both the late and early endosomes. This is in agreement with endosomal ablation studies where it was shown that around 40 % of cellular GLUT4 is found within transferrin receptor positive compartments (Livingstone et al. 1996). The number of immuno-gold particles in the endosomes is reduced to 25 % after stimulation with insulin, suggesting a movement of GLUT4 towards the plasma membrane from this compartment. Intriguingly, prolonged insulin stimulation was found to cause fusing cargo to switch from 60 nm GSVs to larger exocytic vesicles, characteristic of endosomes, therefore suggesting an additional role of the endosomes and endosome-derived structures in GLUT4 translocation (Xu et al. 2011). Furthermore, transferrin and Rab14-positive endosomal vesicles have been shown to fuse with the plasma membrane in response to insulin stimulation, albeit at a slower rate than GSV fusion. Nevertheless, this data suggests that endosomes may play a role in GLUT4 translocation, but the precise details, remain unclear.

An increased number of vesicles were observed within the vicinity of the plasma membrane in the insulin-stimulated state (Figs. 6 and 7). Furthermore, increased GLUT4 labelling of the plasma membrane, coated pits and vesicular tubular elements near the PM was also found. When combined, the total labelling of these structures amounted to 40 % of the total GLUT4 labelling, which is in good concordance with the 38 % and 35 % previously seen in white adipose and brown adipose, respectively (Malide et al. 2000; Slot et al. 1991). Interestingly, insulin has been reported to not only increase the frequency of fusion events, but also to stimulate the docking and tethering of GLUT4 vesicles to the plasma membrane, resulting in an accumulation of GLUT4 vesicles in and around the cell surface (Lizunov et al. 2005). Furthermore, insulin has been shown to result in a switch to internalisation of GLUT4 via a clathrin-mediated pathway. This is therefore supported by the increased labelling of coated vesicles in the vicinity of the plasma membrane in the insulin-stimulated state (Blot and McGraw 2006).

It remains to be determined whether these vesicles at the plasma membrane contain GSV markers and proteins involved in the fusion machinery and whether these coated vesicles represent clathrin-positive structures. Moreover, it remains to be determined, whether the increased numbers of vesicles near the plasma membrane are vesicles primed for fusion or whether they represent vesicles that have fused and are being endocytosed back into the cell. This could be achieved by labelling cells stimulated with insulin, with a fluid phase marker, such as BSA-gold. If the vesicles surrounding the cell surface have fused with the plasma membrane and are internalising into the cell, then they will be labelled with BSA-gold.

Interestingly, the vesicle clusters, suspected to be GSVs, in addition to containing less GLUT4, were not as prevalent in the insulin-stimulated state when examined qualitatively. This suggests that the increase in the number of vesicles within the vicinity of the plasma membrane may be due to a direct translocation of vesicles from the clusters in the juxtanuclear region. Immuno-electron microscopy using antibodies against GSV-resident proteins should be employed to test this hypothesis. If the vesicles within the vicinity of the cell surface represent vesicles that have translocated directly from the GSVs, then they should contain the same complement of proteins.

Conflicting evidence exists as to the involvement of the TGN in GLUT4 trafficking and recycling. Figures 5 and 7 reveal little labelling of vesicles on the *trans* side of the Golgi cisternae in both endogenous and pXLG_3_.HA.GLUT4.GFP expressing cells regardless of the stimulation state. Furthermore, GLUT4 does not co-localise with the TGN resident protein, TGN38 (Martin et al. 1994).

This data however, contradicts previous immuno-electron microscopy studies, which have suggested that between 14 % and 55 % of the total GLUT4 labelling is located within the trans Golgi reticulum (TGR). In addition, it has been suggested that GLUT4 traffics via the TGN in a subdomain enriched in syntaxin 6 and 16 (Shewan et al. 2003). However the morphology of adipocytes is such, that many structures, including the TGN and recycling endosomes are concentrated within the juxtanuclear region of the cell, thus making it difficult to differentiate between them, which therefore may explain the contradictory evidence. The compartment identified in this chapter as vesicles and tubules within the juxtanuclear region of the cell may in fact represent a subdomain of the TGN. It is therefore essential that double labelling with markers of the TGN, such as syntaxin 6 and 16 and markers of the GSVs be performed using the Tokuyasu CLEM technique to correctly identify this compartment.

In conclusion, the stable pXLG_3_.HA.GLUT4.GFP cell line produced by lentiviral transduction displays similar localisation and trafficking to that of endogenous GLUT4 and transiently over expressed HA.GLUT4.GFP. Furthermore a quantitative CLEM assay has been successfully used in order to study the localisation of pXLG_3_.HA.GLUT4.GFP in 3T3-L1 adipocytes. The morphologies of intracellular compartments within the adipocyte have been defined and the amount of GLUT4 within these structures has been quantified. Furthermore, insulin has been shown to promote translocation of GLUT4 from intracellular clusters of vesicles to the plasma membrane. While further work is required to define the protein composition of each compartment and identify their roles in GLUT4 trafficking, the use of CLEM may prove crucial in dissecting the GLUT4 trafficking pathway and identifying key proteins involved in GLUT4 sorting.

## Abbreviations

GLUT4: Glucose transporter type 4
SNARE: SNAP receptor
VAMP2: vesicle associated membrane protein 2
IRAP: Interleukin-1 receptor antagonist protein
HA: Influenza virus hemagglutinin
GFP: Green fluorescent protein
GSV: GLUT4 storage vesicle
CLEM: Correlative Light Electron Microscopy
FRET: fluorescence resonance energy transfer
VSV-G: Vesicular stomatitus virus glycoprotein
PBS: Phosphate buffered saline
BSA: Bovine serum albumin
TGN: Trans Golgi network
TGR: Trans Golgi reticulum
EEA1: Early endosome antigen 1
